# CB2 cannabinoid receptor expression is increased in 129S1/SvImJ mice: behavioral consequences

**DOI:** 10.3389/fphar.2022.975020

**Published:** 2022-08-23

**Authors:** Marc Ten-Blanco, Inmaculada Pereda-Pérez, Cristina Izquierdo-Luengo, Fernando Berrendero

**Affiliations:** Faculty of Experimental Sciences, Universidad Francisco de Vitoria, Madrid, Spain

**Keywords:** anxiety, fear, prepulse inhibition, CB2 cannabinoid receptor, mouse

## Abstract

Genetic and environmental factors are implicated in the etiology of neuropsychiatric diseases. Inbred mouse strains, including the 129S1/SvImJ (S1), constitute important models to study the influence of genetic factors in these conditions. S1 mice displayed anxiogenic-like behavior, impaired fear extinction, and increased prepulse inhibition (PPI) of startle reflex compared to C57BL/6J (BL6) mice. Given the role played by the endocannabinoid system (ECS) in these responses, we evaluated the expression of the ECS components in different brain regions in S1 mice. Gene expression levels of the cannabinoid type-1 and type-2 receptors (CB1R and CB2R) and the endocannabinoid metabolizing enzymes varied depending on the brain region evaluated. Notably, CB2R expression markedly increased in the amygdala, prefrontal cortex and hippocampus in S1 mice. Moreover, CB2R blockade with SR144528 partially rescued the anxiogenic phenotype in S1 mice, while CB2R activation with JWH133 potentiated the deficits in fear extinction and the PPI of startle reflex in this mouse strain. These data suggest that CB2R is involved in the behavioral alterations observed in S1 mice and underline the importance of this cannabinoid receptor subtype in the regulation of certain central nervous system disorders.

## Introduction

Genetic predisposition and environmental factors contribute to the development of psychiatric disorders (Uher, 2014). However, clearly more research is needed to fully understand the causes underlying individual differences in risk and resilience for these diseases, including genetic variation. Diverse genetically inbred mouse strains exist, which represent exceptional models for studying the influence of genetic factors in neuropsychiatric disorders (Moore et al., 2020). In this sense, the inbred 129S1/SvImJ (S1) mouse strain displays poor fear extinction (Hefner et al., 2008), dysregulated hypothalamic-pituitary-adrenal axis function (Camp et al., 2012), behavioral alterations associated with increased stress reactivity (Rodriguez et al., 2020), and sleep disturbances (Fritz et al., 2021). Therefore, this strain may represent a useful model to elucidate distinct and overlapping mechanisms underlying different maladaptive behaviors.

Considering the range of possible neurobiological mechanisms involved in the S1 mice phenotype, the endocannabinoid system (ECS), composed of two main receptors, the cannabinoid type-1 and type-2 receptors (CB1R and CB2R, respectively), their ligands, i.e., the endocannabinoids anandamide (AEA) and 2-arachidonoylglycerol (2-AG), and the enzymes involved in endocannabinoid metabolism (Mechoulam and Parker, 2013) could be a promising candidate. This neuromodulatory system plays a crucial role in different neurophysiological processes. Disturbances in the ECS, mainly related to CB1R dysfunction, are associated with several psychiatric conditions such as posttraumatic stress disorder (Mayo et al., 2022), anxiety (Petrie et al., 2021) or schizophrenia (Leweke et al., 2018), among others. Interestingly, CB2R, initially regarded as a peripheral cannabinoid receptor, has been recently involved in the regulation of different neurobiological processes including cognition and mood-related (anxiety, depression) behaviors (Banaszkiewicz et al., 2020). In agreement with potential modifications of the ECS as a molecular mechanism contributing to the phenotypic alterations observed in S1 strain, the selective fatty acid amide hydrolase (FAAH) inhibitor AM3506 rescued fear extinction deficits in these mice (Gunduz-Cinar et al., 2013) by increasing AEA levels in the amygdala. This effect was dependent on CB1R activation in this brain region since the fear-reducing effects of systemic AM3506 were blocked by intra-amygdala infusion of the CB1R antagonist rimonabant (Gunduz-Cinar et al., 2013).

The aim of this study was to analyze the expression of the main components of the ECS in several brain areas of S1 strain compared to C57BL/6J (BL6) mice. Considering the main change observed, we also evaluated the consequences of the modulation of CB2R in S1 mice in key neurobehavioral responses such as anxiety, fear conditioning and extinction, and sensorimotor gating.

## Material and methods

### Animals

Experiments were performed using male 129S1/SvImJ (S1) mice (Jackson Laboratories) and C57BL/6J (BL6) mice (Charles River) at 8–10 weeks old. BL6 mice were chosen as the comparison strain in this study because they represent one of the most commonly used mouse lines in neuroscience research. Moreover, previous work evaluating fear extinction in the S1 strain typically used BL6 mice as a reference, since they exhibit proper fear extinction, acquisition and recall (Rodriguez et al., 2020). Mice were housed by strain (maximum 5 per cage) and maintained in a temperature (21.1 ± 1°C)- and humidity (55% ± 10%)-controlled room under a 12-h light/dark cycle (lights on at 8:00 a.m.). Food and water were available *ad libitum*. All experiments were performed during the light phase. Mice were handled daily for 3 days before the beginning of the experiment. Experimental procedures were conducted in the animal facilities of Universidad Francisco de Vitoria in Madrid, Spain, in accordance with the guidelines of the European Communities Directive 2010/63/EU and the Spanish Regulations RD 1201/2005 and 53/2013 regulating animal research and approved by the local ethical committee (CEEA-UFV).

### Drugs

The CB2R agonist JWH133 (5 mg/kg) (Tocris) was dissolved in a solution of 10% DMSO, 10% Tween 80 and 80% saline. The CB2R antagonist SR144528 (3 mg/kg) (Sigma) was dissolved in a solution of 5% ethanol, 5% cremophor and 90% saline. Both drugs were administered by intraperitoneal (ip) route (10 ml/kg body weight). Doses were based on previous studies in mice (Busquets-Garcia et al., 2011; Donvito et al., 2017) in mice.

### Elevated plus maze test

Anxiety-like behavior was assessed by using a black maze elevated 30 cm above the ground with four arms (25 cm × 5 cm) set in a cross from a neutral central square (5 cm × 5 cm). Two opposite arms were delimited by walls (closed arms) and illuminated with 4–6 lux, whereas the two other opposite arms had unprotected edges (open arms) and were illuminated with 40–50 lux. Pharmacological treatments were administered 30 min before the test. The total number of visits to the closed and open arms, and the cumulative time spent in each arm were observed through a videocamera system during 5 min.

### Cued fear conditioning and extinction

Mice were cued fear-conditioned as performed in preceding experiments with slight modifications (Flores et al., 2014). The test chamber (LE116, Panlab, Harvard Instruments) was made with black methacrylate walls and a transparent front door. This chamber (25 cm × 25 cm × 25 cm) was located inside a soundproof module with a ventilation fan in order to provide a background noise and attenuate surrounding sounds. The chamber floor was constructed of parallel stainless-steel bars of 2 mm of diameter spaced at 6 mm intervals and was connected to a scrambled shock generator (LE100-26 module, Panlab, Harvard Instruments). A high-sensitivity weight transducer (load cell unit) was used to record and analyze the signal generated by the animal movement intensity. Experimental software PACKWIN V2.0 automatically calculated the percentage of immobility time for each experimental phase. Before each trial, the chamber floor and walls were cleaned with 70% ethanol and then water to avoid olfactory cues. On the conditioning session, mice were individually placed in the chamber during 180 s before the onset of three cue tones (3 kHz, 90 dB, 30 s long, 10 s between tones), each one co-terminating with a footshock (0.7 mA, 1 s). After the last cue tone, mice remained in the chamber for 10 s. Fear extinction sessions (E1–E5) were performed 24, 48, 72, 96 and 120 h after the conditioning day in a different context (transparent Plexiglas cylinder surrounded by white walls and a smooth floor). In E1, mice were habituated to the new context during 180 s, whereas in E2–E5 this habituation time was reduced to 60 s. After the habituation, mice were re-exposed to the CS (4 cue tones, 30 s long, 10 s between tones). To study the fear extinction process, pharmacological treatments were administered 30 min before each extinction session. Fear memory was assessed as the percentage of time that mice spent freezing during the 4 cue tones of each extinction session. Freezing behavior, a rodent’s natural response to fear, was automatically evaluated and defined as complete lack of movement, except for breathing for more than 800 ms. Data from fear extinction were expressed as percentage of freezing behavior and as area under the curve (AUC). AUC was calculated by using a standard trapezoid method, AUC = [0.5 × (B1 + B2) × h] + [0.5 × (B2 + B3) × h] + … [0.5 × (Bn + Bn + 1) × h], where Bn were the percentage of freezing behavior for each mouse and h was the time (days) passed between the consecutive measurements.

### Prepulse inhibition of startle reflex

Prepulse inhibition (PPI) of startle reflex, a measure of sensorimotor gating, was assessed by using the StartFear Combined System (Panlab, Harvard Instruments). Mice were daily habituated to a Plexiglas cylinder located inside the sound-attenuating chamber for 5 min with background white noise (65 dB) 4 days prior to the test. The test started with 5 min habituation in the cylinder and, immediately after, mice were exposed to 5 pulse trials (120 dB, white noise, 40 ms). These trials were performed for startle accommodation and were excluded in the final analysis. The experimental protocol consisted of 10 blocks with 6 or 12 trials each, randomly presented to mice with an inter-trial interval of 7–23 s. Blocks consisted of: no stimulus (6×) (background white noise), pulse alone (12×) (120 dB, white noise, 40 ms), pulse preceded by 4 prepulse intensities (12× each) (4, 8, 12 and 16 dB above background noise, 20 ms, 100 ms before pulse) and the prepulses alone (6× each). A background white noise was generated throughout the whole experiment. Pharmacological treatments were administered 30 min before the test. Startle amplitude was detected by PACKWIN V2.0 software. Percent PPI was calculated as follows: 100 × (startle response – prepulse inhibited startle response) / startle response.

### Quantitative RT-PCR analysis

Amygdala, prefrontal cortex and hippocampus tissues were extracted in basal conditions and immediately frozen at −80°C. These brain areas were chosen based on their implication in the behavioral responses evaluated in this study. Total RNA was purified with the RiboPure™ Kit (Invitrogen) for amygdala and prefrontal cortex, and the RNeasy Mini Kit (QIAGEN) for hippocampus, according to the manufacturer’s instructions. Reverse transcription was performed with 0.9 μg of total RNA and the SuperScript™ II Reverse Transcriptase (Invitrogen). PCR reactions were conducted using PrimePCR™ Probe Assay (Bio-Rad) to quantify mRNA levels for: CB1R (Unique Assay ID: qMmuCEP0038879), CB2R (Unique Assay ID: qMmuCEP0039299), DAGLα (Unique Assay ID: qMmuCIP0032590), MAGL (Unique Assay ID: qMmuCIP0042348), NAPE-PLD (Unique Assay ID: qMmuCIP0035707) and FAAH (Unique Assay ID: qMmuCEP0055480), using GAPDH expression (Unique Assay ID: qMmuCEP0039581) as endogenous control gene for normalization. PCR assays were carried out with the CFX Connect Real-Time PCR Detection System (Bio-Rad). The fold changes in gene expression of S1 in comparison with BL6 mice were calculated using the 2^−ΔΔCt^ method.

### Statistical analysis

Comparisons between two groups were assessed by Student’s *t* tests. Multiple-group comparisons were performed by one-way analysis of variance (ANOVA). Repeated-measurement ANOVA was used for serial freezing responses and startle amplitude response between the different prepulse intensities. Subsequent Fisher’s LSD post-hoc test was only used when ANOVA interaction effects were significant. All data were expressed as mean ± SEM. The statistical analysis was performed using Statistica (StatSoft) software. The level of significance was *p* < 0.05 in all experiments.

## Results

### Anxiogenic-like behavior, impaired fear extinction, and increased prepulse inhibition of startle reflex in S1 mice

**FIGURE 1:**
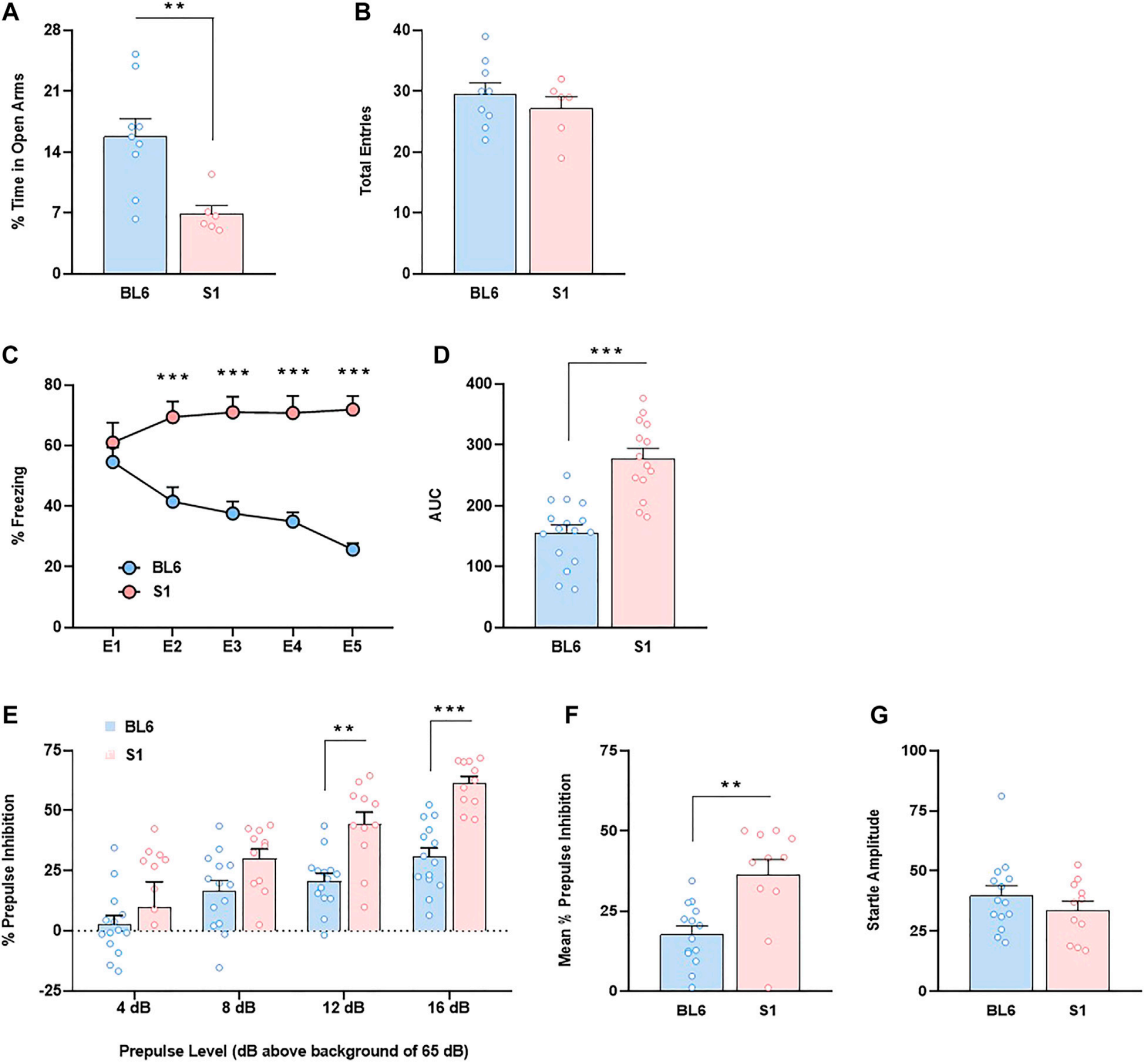
Anxiety-like behavior, impaired fear extinction and increased prepulse inhibition in S1 compared to BL6 mice. **(A,B)** Percentage of time spent in the open arms **(A)** and total number of entries in both closed and open arms **(B)** of the elevated plus maze test (*n* = 6–9 mice per group). **(C,D)** Time course **(C)** and AUC values **(D)** of the freezing levels during cued fear extinction trials (*n* = 14–16 mice per group). **(E–G)** Percentage of prepulse inhibition **(E)**, mean of the percentage of prepulse inhibition **(F)** and startle response amplitude **(G)** (*n* = 11–14 mice per group). Data are expressed as mean ± SEM. *p < 0.05, **p < 0.01, ***p < 0.001 (comparison between BL6 and S1). BL6: C57BL/6J mice strain; S1: 129S1/SvImJ mice strain; E1–E5: extinction trials 1–5; AUC: area under the curve.

First, we carried out a direct comparison between S1 and BL6 mice in several neurobehavioral responses. Unconditioned anxiety was evaluated by using the elevated plus maze (EPM). S1 mice showed an anxiogenic-like effect (*p* < 0.01) (Figure 1A) as revealed the decrease of the percentage of time spent in open arms. No changes were observed in the total number of entries (Figure 1B). Cued fear conditioning was not modified in S1 mice as showed similar freezing behavior between S1 and BL6 strains in the E1 session (Figure 1C). However, as previously reported (Hefner et al., 2008; Whittle et al., 2010), fear extinction was impaired in S1 mice as revealed the increase of freezing behavior (F_4,116_ = 8.95, *p* < 0.001) and area under the curve (AUC) (*p* < 0.001) (Figures 1C,D) when compared to BL6 strain. Then, we performed the PPI test to study effects on sensorimotor gating. S1 mice showed a significant increase in basal PPI (F_3,69_ = 3.43, *p* < 0.05) in comparison with BL6 mice (Figure 1E). This effect was significant at the prepulses of 12 (*p* < 0.01) and 16 dB (*p* < 0.001) above background of 65 dB (Figure 1E). The mean PPI score was ∼51% higher in S1 than in BL6 mice (*p* < 0.01) (Figure 1F). This effect was independent of baseline changes in startle amplitude (Figure 1G), discarding an impact of startle reaction in the PPI modifications observed.

### Increased CB2 cannabinoid receptor expression in the amygdala, prefrontal cortex and hippocampus in S1 mice

**FIGURE 2:**
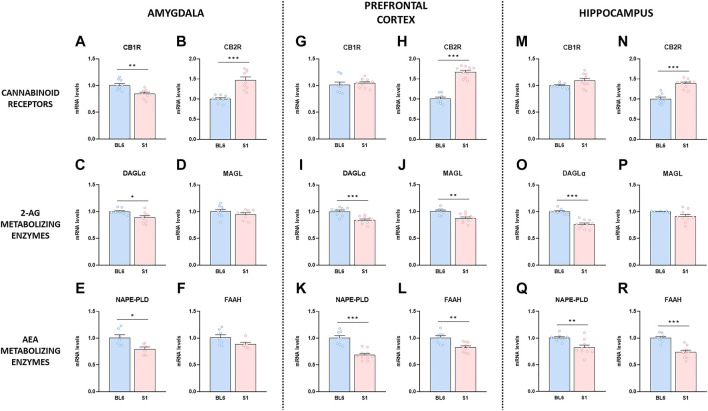
Changes in gene expression of the endocannabinoid system compounds in the S1 strain compared with BL6. Gene expression levels of the CB1R and CB2R, the 2-AG metabolizing enzymes DAGLα and MAGL, and the AEA metabolizing enzymes NAPE-PLD and FAAH in amygdala **(A–F)**, prefrontal cortex **(G–L)** and hippocampus **(M–R)** in BL6 and S1 mice (*n* = 6–10 mice per group). Data are expressed as mean ± SEM. *p < 0.05, **p < 0.01, ***p < 0.001 (comparison between BL6 and S1). BL6: C57BL/6J mice strain; S1: 129S1/SvImJ mice strain.

Given the role played by the ECS in the regulation of the behavioral responses altered in S1 mice, we evaluated basal gene expression of CB1R and CB2R, and the endocannabinoid-synthesizing and degrading enzymes in this mouse strain. S1 mice presented lower gene expression level of CB1R in the amygdala (*p* < 0.01) (Figure 2A), without changes either in the prefrontal cortex (Figure 2G) or the hippocampus (Figure 2M) compared to BL6 mice. Notably, quantitative RT-PCR analysis showed a robust increase of CB2R mRNA levels in the amygdala (∼47%) (*p* < 0.001) (Figure 2B), prefrontal cortex (∼67%) (*p* < 0.001) (Figure 2H), and hippocampus (∼39%) (*p* < 0.001) (Figure 2N) in S1 mice. The expression of the enzyme in charge of 2-AG synthesis DAGLα was significantly decreased in the amygdala (*p* < 0.05) (Figure 2C), prefrontal cortex (*p* < 0.001) (Figure 2I), and hippocampus (*p* < 0.001) (Figure 2O), while MAGL expression (enzyme that degrades 2-AG) was only reduced in the prefrontal cortex (*p* < 0.01) in S1 strain (Figure 2J), with no differences in the amygdala (Figure 2D) and the hippocampus (Figure 2P). Finally, we analyzed the mRNA levels of NAPE-PLD and FAAH, the enzymes responsible for the synthesis and degradation of AEA, respectively. A decrease in the expression of NAPE-PLD was found in the three brain regions evaluated (amygdala, *p* < 0.05, Figure 2E; prefrontal cortex, *p* < 0.001, Figure 2K; hippocampus, *p* < 0.01, Figure 2Q). The expression of FAAH was significantly decreased in the prefrontal cortex (*p* < 0.01) (Figure 2L) and the hippocampus (*p* < 0.001) (Figure 2R), while no differences were observed in the amygdala (Figure 2F) in S1 mice.

### Pharmacological modulation of CB2 cannabinoid receptors triggers behavioral changes in S1 mice

**FIGURE 3:**
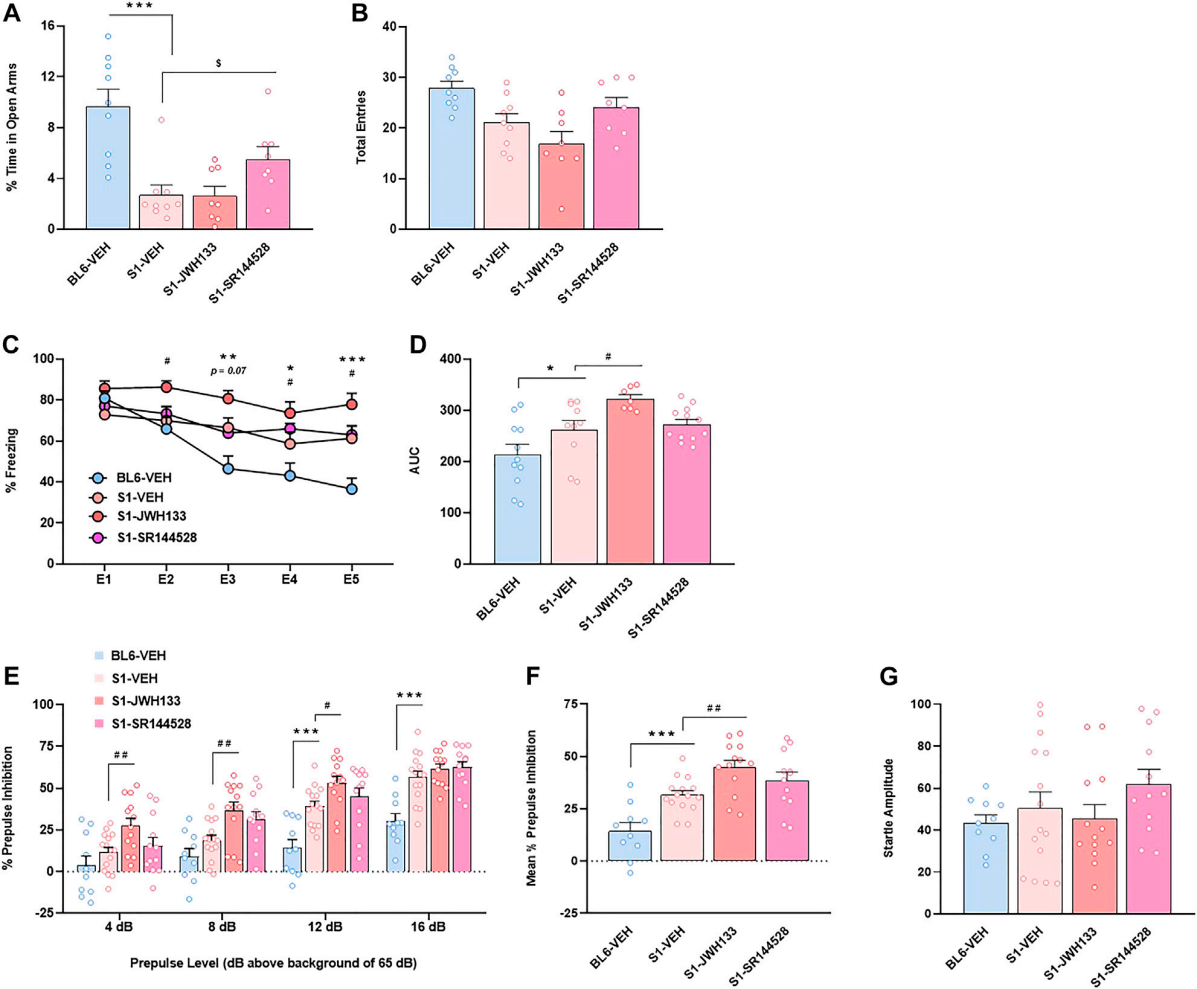
CB2R is involved in the anxiety-like behavior, fear extinction deficits and increased prepulse inhibition response of the S1 mice strain compared to BL6 mice. **(A,B)** Percentage of time spent in the open arms **(A)** and total number of entries in both closed and open arms **(B)** of the elevated plus maze test in BL6 and S1 mice treated with the CB2R agonist JWH133 (5 mg/kg, ip) or the CB2R antagonist SR144528 (3 mg/kg, ip) 30 min before the test (*n* = 8–9 mice per group). **(C,D)** Time course **(C)** and AUC values **(D)** of the freezing levels during cued fear extinction trials in BL6 and S1 mice treated with JWH133 (5 mg/kg, ip) or SR144528 (3 mg/kg, ip) 30 min before each extinction session (*n* = 7–12 mice per group). **(E–G)** Percentage of prepulse inhibition **(E)**, mean of the percentage of prepulse inhibition **(F)** and startle response amplitude **(G)** in BL6 and S1 mice treated with JWH133 (5 mg/kg, ip) or SR144528 (3 mg/kg, ip) 30 min before the test (*n* = 10–15 mice per group). Data are expressed as mean ± SEM. *p < 0.05, **p < 0.01, ***p < 0.001 (comparison between BL6-VEH and S1-VEH); ^$^ *p* < 0.05 (comparison between S1-VEH and S1-SR144528); ^#^ *p* < 0.05, ^##^ *p* < 0.01 (comparison between S1-VEH and S1-JWH133). BL6: C57BL/6J mice strain; S1: 129S1/SvImJ mice strain; E1-E5: extinction trials 1–5; AUC: area under the curve.

In view of the unexpected and strong basal increased expression of CB2R in S1 strain, we studied the consequences of the modulation of this cannabinoid receptor subtype in the phenotypic alterations previously observed in these mice. The acute administration of the CB2R antagonist SR144528 partially prevented the anxiogenic phenotype of S1 mice in the EPM test, as revealed one-way ANOVA (F_3,30_ = 11.81, *p* < 0.001) and post hoc comparison between S1 groups treated with vehicle or SR144528 (*p* < 0.05) (Figure 3A). No modification was observed in the total number of entries between these two groups (Figure 3B). Moreover, the injection of the CB2R agonist JWH133 did not alter anxiety-like behavior in S1 mice (Figure 3A). In contrast, JWH133 potentiated the resistance of cued fear extinction in S1 mice as showed the increase of freezing behavior (F_12,144_ = 3.73, *p* < 0.001) (*p* < 0.05 at E2, E4 and E5) and AUC (F_3,36_ = 7.05, *p* < 0.001) (*p* < 0.05) (Figures 3C,D) when compared to S1 mice treated with vehicle. However, the administration of SR144528 did not modify fear extinction in S1 mice (Figures 3C,D). A significant increase of PPI of startle reflex was observed by the administration of JWH133 in S1 strain (F_9,138_ = 2.70, *p* < 0.01) (Figure 3E). Thus, post hoc comparison revealed differences between S1 mice treated with vehicle or JWH133 at the prepulses of 4 (*p* < 0.01), 8 (*p* < 0.01), and 12 dB (*p* < 0.05) above background of 65 dB (Figure 3E). An overall increase of PPI due to JWH133 treatment was observed when representing mean PPI score (F_3,46_ = 13.07, *p* < 0.001) (*p* < 0.01) (Figure 3F). However, the magnitude of startle reflex was not altered by JWH133 injection (Figure 3G). SR144528 administration did not modify PPI behavior (Figures 3E,F) nor startle amplitude (Figure 3G) in S1 mice. Taken together, these results suggest that CB2R could take part of the molecular mechanisms that underlie the phenotypic alterations of the S1 strain.

## Discussion

Our data show remarkable changes in the expression levels of several components of the ECS in different brain regions in S1 mice. Particularly interesting, CB2R expression was strongly increased in the amygdala, prefrontal cortex and hippocampus in this strain compared to BL6 mice. These alterations suggest that CB2R could be involved in the phenotypic characteristics observed in S1 mice. Indeed, acute pharmacological modulation of CB2R induced behavioral alterations in important neurobiological processes in these mice. Future experiments evaluating the effects of chronic CB2R agonists and antagonists in S1 mice would be interesting since acute or chronic administration of CB2R ligands could result in different responses (García-Gutiérrez et al., 2012). The use only of male mice is a limitation of this study as several reports show evidences for sex differences in animal models of neurobehavioral disorders (Palanza and Parmigiani, 2017).

Genetic differences between strains are likely to affect several phenotypic features offering a powerful tool with which to expand our knowledge about the factors that influence psychiatric conditions. S1 inbred mice showed higher innate anxiety compared to BL6 mice as revealed the decrease of the percentage of time in open arms in the EPM test. In agreement, these mice spent significantly less time in the center of the open field (Rodriguez et al., 2020) and in the light compartment in the light-dark box (Millstein and Holmes, 2007) confirming an anxiogenic-like behavior. Cued fear extinction was impaired in S1 mice relative to the good-extinguishing BL6 strain, as previously established (Hefner et al., 2008; Whittle et al., 2010). Finally, we observed an increase of PPI of startle reflex in S1 compared to BL6 mice without modification of the startle amplitude. A high level of PPI was previously seen in S1 mice in relation to BL6 strain (Millstein et al., 2006), although no direct comparison between strains was performed in this study. Considering that the ECS participates in the regulation of anxiety (Petrie et al., 2021), extinction of aversive memories (Mizuno and Matsuda, 2021) and sensorimotor gating (Dissanayake et al., 2013), this neuromodulatory system could contribute to the phenotypic alterations observed in S1 mice. Interestingly, we found important differences in the mRNA levels of various components of the ECS between S1 and BL6 mice in several brain areas.

Our results showed a significant reduction of CB1R and NAPE-PLD expression in the amygdala of S1 strain which could in part explain the anxiogenic phenotype and resistance of fear extinction characteristic of these mice. In agreement, CB1R knockout mice are anxiogenic (Martin et al., 2002) and show strongly impaired extinction in auditory fear-conditioning tests (Marsicano et al., 2002). The recently developed NAPE-PLD inhibitor LEI-401 reduced AEA levels in the mouse brain and impaired extinction of an aversive memory in BL6 mice (Mock et al., 2020). Moreover, the systemic administration of the selective FAAH inhibitor AM3506 rescued fear extinction deficits in S1 mice (Gunduz-Cinar et al., 2013). This effect was fully recapitulated by intra-amygdala infusion of AM3506 by a mechanism involving CB1R (Gunduz-Cinar et al., 2013), consistent with the changes observed in our study. On the other hand, the expression of DAGLα also decreased in the amygdala in S1 strain. This change could be also related to the extinction deficits typical of this strain since mice deficient in DAGLα, which have reduced 2-AG brain levels, also exhibit impaired fear extinction (Jenniches et al., 2016). DAGLα, NAPE-PLD and FAAH expression were also reduced in the prefrontal cortex and hippocampus in S1 mice, brain areas which are also important mediators of fear regulation and anxiety (Maren et al., 2013). Future studies evaluating endocannabinoid levels will help to clarify the possible functional relevance of the changes in the expression of these enzymes in S1 strain.

Interestingly, CB2R expression was strongly enhanced in the amygdala, prefrontal cortex, and hippocampus in S1 related to BL6 mice. The increased expression of CB2R could be the result of a compensation of the general reduced expression of the synthesizing and metabolizing enzymes and associated possible changes in endocannabinoid levels in S1 mice. This is the case of other neurotransmission systems such as the opioid system. Thus, marked region-specific up-regulation of the mu, delta, and kappa opioid receptors was observed in mice lacking the proenkephalin and prodynorphin genes (Clarke et al., 2003). In any case, future experimental work will be necessary to establish a possible relationship between the changes in enzymes gene expression and the increased CB2R expression observed in S1 mice. Although CB2R was initially considered as a peripheral cannabinoid receptor, several recent studies suggest a role of this receptor in several neuropsychiatric disorders (Banaszkiewicz et al., 2020; Kibret et al., 2022). Contradictory results have been obtained regarding the potential function of CB2R in the regulation of anxiety-like behavior in both genetic and pharmacological studies. Deletion of the CB2R produced an anxiogenic-like response in the EPM test (Ortega-Alvaro et al., 2011), while CB2R conditional knockout mice in dopamine neurons showed an anxiolytic-like phenotype in the same test (Liu et al., 2017). CB2R overexpression in mice decreased vulnerability to anxiety (García-Gutiérrez and Manzanares, 2011), but induced an impairment of the anxiolytic action of alprazolam. Acute treatment with the CB2R antagonist AM630 increased anxiety in Swiss ICR mice (García-Gutiérrez et al., 2012), while the administration of the CB2R agonist JWH133 did not produce any effect in the same study. Our data suggest that the increased expression of CB2R found in the amygdala, prefrontal cortex and hippocampus (brain areas involved in regulating anxiety-like behaviors) in S1 mice could be related to the anxiogenic phenotype of these mice. Thus, the acute administration of the CB2R antagonist SR144528 partially rescued the anxiogenic-like behavior in S1 mice. In this sense, chronic treatment with AM630 reduced anxiety-like behavior in the spontaneously anxious DBA/2J strain of mice (Yilmazer-Hanke et al., 2003), suggesting that this cannabinoid receptor may result a relevant target for the treatment of anxiety-like disorders. Acute effects of AM630 in anxiety were not evaluated in DBA/2J mice in this study (García-Gutiérrez et al., 2012).

S1 mice constitute a well-established model of impaired fear extinction (Hefner et al., 2008; Whittle et al., 2010). Recently, a novel role of amygdalar CB2R in the regulation of the extinction of aversive memories has been reported in mice (Ten-Blanco et al., 2022). The intra-amygdala infusion of the CB2R antagonist AM630 blocked the fear extinction deficits induced by the overactivation of the orexin/hypocretin system, while the systemic administration of the CB2R agonist JWH133 promoted fear extinction resistance (Ten-Blanco et al., 2022). Therefore, the increase in CB2R mRNA levels observed in three prototypical areas regulating fear (i.e., amygdala, prefrontal cortex and hippocampus) in S1 mice could contribute to the impaired fear extinction characteristic of this strain. Indeed, JWH133 potentiated the extinction deficits in S1 mice, although the CB2R antagonist SR144528 did not affect this behavioral response. Congruent with this, fear extinction in S1 strain was not improved by systemic treatment with the NMDA receptor partial agonist d-cycloserine, known to facilitate extinction in rodents and effective as an adjunct to exposure therapy in human anxiety disorders (Davis et al., 2006). The inefficacy of d-cycloserine or SR144528 in S1 mice could reflect usually complex molecular mechanisms driving the extinction behavior in this mouse strain (Hefner et al., 2008).

PPI of the startle reflex is a sensorimotor gating process that reduces the startling response when a weaker sensory stimulus precedes a sudden startling stimulus. An impairment in the PPI response is observed in patients with schizophrenia (Mena et al., 2016). Several studies present evidence of the contribution of CB2R to the modulation of sensorimotor gating. Thus, AM630 potentiated the reduction of PPI induced by both the NMDA receptor antagonist MK801 and methamphetamine (Ishiguro et al., 2010), although the CB2R antagonist did not affect PPI on its own. In contrast to the pharmacological data, CB2R knockout mice showed disrupted PPI at different prepulse intensities (Ortega-Alvaro et al., 2011). On the other hand, MK801-induced decrease in PPI was attenuated by the CB2R agonists JWH015 (Khella et al., 2014) and HU-910 (Cortez et al., 2022). These results are congruent with an involvement of CB2R in the enhanced PPI observed in S1 mice. The expression of CB2R was dramatically increased in the prefrontal cortex of S1 strain, a brain area directly related to the modulation of sensorimotor gating (Tóth et al., 2017). Moreover, the administration of the CB2R agonist JWH133 enhanced the PPI inhibition in S1 mice in comparison with S1 treated with vehicle at different prepulse intensities.

In summary, our data reveal important changes in the expression levels of different components of the ECS in S1 mice. Particularly significant, the increased expression of CB2R observed in this mouse strain could contribute to its behavioral alterations. Elucidating the CB2R cell type location would shed light on the role of this cannabinoid receptor in the different functions described above. However, CB2R antibody nonspecificity strongly hinders its identification (Atwood and Mackie, 2010). CB2R antagonism with SR144528 partially rescued the anxiogenic-like behavior in S1 mice. The CB2R agonist JWH133 did not enhance anxiety in S1 mice possible due to a ceiling effect, as the percentage of time in open arms was very low in these animals. On the other hand, the CB2R agonist JWH133 worsened fear extinction and enhanced PPI of startle reflex. SR144528 did not improve fear extinction or decrease PPI in S1 mice probably because of complex molecular mechanisms driving the extinction behavior or the PPI in this mouse strain, as previously suggested (Hefner et al., 2008). Our study also underlines a role for the CB2R in the regulation of important functions of the central nervous system.

## Data Availability

All data generated or analysed during this study are available from the authors on reasonable request.
